# Nonlinear Ultrasonic Imaging for Porosity Evaluation

**DOI:** 10.3390/s23146319

**Published:** 2023-07-12

**Authors:** Mario Emanuele De Simone, Salvatore Boccardi, Gian Piero Malfense Fierro, Michele Meo

**Affiliations:** 1 Department Aeronautics and Astronautics, University of Southampton, Southampton SO17 1BJ, UK; 2 MTC Limited, Ansty Business Park, Coventry CV7 9JU, UK

**Keywords:** porosity, ultrasound, nonlinear ultrasound, phased array, imaging, nonlinear imaging, attenuation

## Abstract

The influence of porosity on the mechanical behaviour of composite laminates represents a complex problem that involves many variables. Therefore, the evaluation of the type and volume content of porosity in a composite specimen is important for quality control and for predicting material behaviour during service. A suitable way to evaluate the porosity content in composites is by using nonlinear ultrasonics because of their sensitivity to small cracks. The main objective of this research work is to present an imaging method for the porosity field in composites. Two nonlinear ultrasound techniques are proposed using backscattered signals acquired by a phased array system. The first method was based on the amplitude of the half-harmonic frequency components generated by microbubble reflections, while the second one involved the frequency derivative of the attenuation coefficient, which is proportional to the porosity content in the specimen. Two composite samples with induced porosity were considered in the experimental tests, and the results showed the high accuracy of both methods with respect to a classic C-scan baseline. The attenuation coefficient results showed high accuracy in defining bubble shapes in comparison with the half-harmonic technique when surface effects were neglected.

## 1. Introduction

In the last few decades, composite materials have been widely used in aeronautic and civil fields due to their high strength and lightweight characteristics. Despite these advantages, composites reinforced with continuous fibres can be affected by the presence of porosity, defined as the concentration of microscopic interfacial voids located in the matrix between plies of fibres and scattered through the thickness of the composite laminate [1]. The presence of porosity in composites may be due to several causes, such as the material constituents and the manufacturing process. In many cases, insufficient pressure during the curing is the most prevalent factor that affects the formation of porosity in a composite laminate [1]. It is well-known that porosity has detrimental effects on the strength of composite specimens, especially with regard to the material’s inter-laminar shear strength [2,3,4,5], compressive strength [6], and bending strength [7], which are associated with matrix-dominated mechanical properties. It has also been shown that these voids result in a measurable decrease in fatigue life [7]. In general, the influence of voids on the mechanical behaviour of composite laminates represents a complex problem that involves a large number of variables, such as shape, size and location of voids; mechanical properties of fibre, matrix and interface; and mechanical loads present and their nature (static or cyclic) [8]. Moreover, it should take into account several environmental factors, such as temperature and moisture [4]. Therefore, the evaluation of the type and volume content of porosity in a composite specimen is essential in the prediction of material behaviour during the service of the specimen and in evaluating its useful life. Current research has focused on the reduction of porosity during composite manufacturing [9], with the assessment of porosity during service largely neglected. Thus, the focus of this work is the non-destructive evaluation of porosity during the service life of composite materials using an ultrasonic technique. Ultrasonic methods are currently the most widely used non-destructive techniques for the quantitative evaluation of porosity in composites because of their simplicity and high sensitivity to the presence of voids. Indeed, there are many previous theoretical and experimental works that explore the dependence of the ultrasonic wave speed and attenuation in composite laminates on the amount of porosity within that same specimen [10,11,12,13,14,15,16,17].

It should be noted that other non-destructive methods involving X-ray computed tomography [18] and thermography [19], or destructive methods based, for example, on acid digestion [20], are not considered in this paper.

Among all the ultrasound techniques, the so-called “linear” methods have shown a high level of accuracy for the detection and localisation of damage in composites; nevertheless, they lack sensitivity in the detection of micro-damage, such as porosity. In the latter case, the creation of nonlinear elastic effects, such as higher harmonics and subharmonics of the excitation frequency, can be used as a signature for micro-damage detection. Therefore, to overcome the limitation of the linear methods in porosity estimation, a nonlinear approach is followed in this paper.

Currently, there are limited methods available for the investigation of porosity in composite materials. These include traditional methods, such as optical microscopy, where cross-sectional samples are inspected under a microscope, and image-based algorithms are used to identify voids; this has limited application due to its destructive nature. X-ray and Neutron Imaging provide a non-destructive alternative where the inspected volumes can be reconstructed and analysed to identify porosity [21,22]. Finally, laser ultrasonic techniques can be used to induce wave propagation within the material, providing information regarding subsurface porosity [23,24,25]. These methods have some limitations regarding equipment cost, inspection speed, and size.

Early work done by Ashwell et al. [26] and Lauterborn [27] showed that nonlinear oscillations of gas bubbles forced into volume pulsations by an acoustic field in liquids might occur at frequencies equal to half of the driving frequency, the subharmonic. Furthermore, Leighton et al. [23] showed that it is possible to accurately size and resolve the bubbles in liquids by interrogating the subharmonic emitted by the bubble, which can be directly related to the resonance of these bubbles. It has also been shown that nonlinear responses occur at the subharmonic as well as at sum and difference frequencies when two frequencies are used to excite bubbles [28,29]. Research into this area of nonlinear ultrasonics is driven by the increased sensitivity to damage and damage evolution, predicted at a few orders of magnitude higher compared with linear techniques, which are only sensitive to gross defects rather than micro-damage [30,31,32]. Nonlinear ultrasound methods can be broken into a few general methods, which include single-frequency excitation, modulation (multiple-frequency mixing), and harmonic generation (including subharmonics) [33,34,35,36,37,38,39]. Among them, nonlinear elastic wave spectroscopy (NEWS) [40,41,42], nonlinear elastic wave modulation spectroscopy (NEWMS) [43,44,45], nonlinear imaging methods [46,47,48], and phase symmetry analysis (PSA) techniques [49,50] were used to explicitly interrogate the nonlinear behaviour of the medium and its effect on wave propagation. Many studies have shown that nonlinearities are generated in structures in the presence of ruptures, voids, cohesive bonds, and the opening/closing of surfaces or micro-cracks [51,52,53]. Material nonlinearity of damaged regions, in terms of nonlinear harmonic generation (due to ultrasound excitation), has been widely used over the past few years as a reliable signature for material flaw detection in both metallic and composite structures [46,54,55].

The aim of this research work was to evaluate induced porosity in two undamaged composite specimens using two different nonlinear ultrasound approaches. The first evaluates and correlates the production of subharmonic responses to porosity, while the second takes into account the attenuation coefficient of the medium. It is expected that gas trapped within resin resonates at specific frequencies to provide subharmonic content in the captured signal, which can then be used to analyse porosity. The first step in this approach is to provide evidence that subharmonic generation can be achieved with porosity, with future work focused on sizing. The two techniques showed different advantages and disadvantages; however, both were able to visualise levels of porosity within the tested composite samples. It should be noted that this work looks to incorporate multiple techniques to increase the probability of the detection of porosity rather than relying on a single method.

The outline of this research work is as follows: the first part of Section 2 describes the nonlinear ultrasound approach and presents both the theory and applications of a standard phased array system. The subharmonic and attenuation coefficient techniques are illustrated, respectively, in Section 2.1 and Section 2.2. Section 3 shows the setup used to perform the experimental tests, while in Section 4, all the results are illustrated. The conclusions of this paper are reported in Section 5.

## 2. Nonlinear Ultrasound Approach

As aforementioned in Section 1, the best approach to extract porosity and void information in a composite specimen is by using nonlinear ultrasound techniques. Therefore, in this paper, two nonlinear ultrasound approaches were developed and investigated to produce a porosity map of the tested composite samples. These maps were compiled by the application of the developed theory on the backscattered signals sent and acquired by a phased array system.

Nonlinear ultrasound phased array techniques have been assessed by many authors. Ohara et al. [56] and Park et al. [57] have extensively developed, evaluated, and improved the detection of open and closed cracks in metallic structures using a subharmonic phased array, while Potter et al. [58] have developed a nonlinear array based on the traditional TFM. These methods provide nonlinear ultrasound information through the thickness of the sample, rely on short excitation signals, have been assessed on metallic structures, and do not generally address issues of equipment-based nonlinearities and porosity.

**Figure 1:**
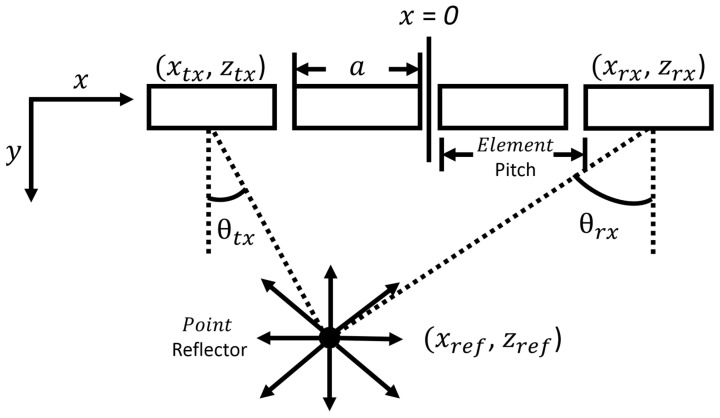
Phased array basic setup geometry.

**Figure 2:**
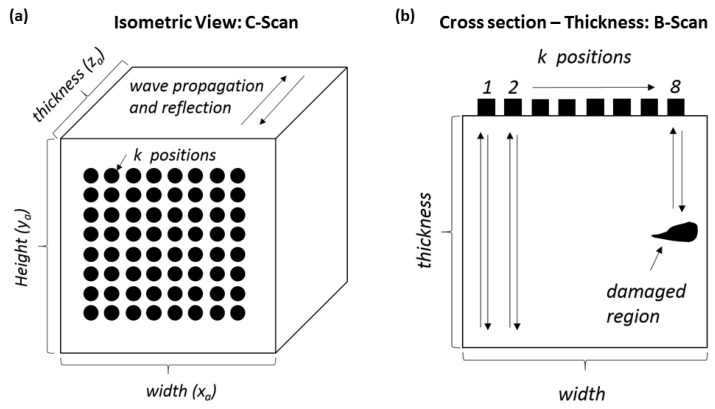
Isometric (**a**) and cross-sectional (**b**) view of scan points for an array setup.

Ultrasonic phased array techniques arguably lead the field in terms of damage detection capabilities and suitability for composite material structures. Phased array systems generally use an array of transmitting and receiving piezoelectric elements, where the sequence of firing and capturing determines the method and accuracy. These systems generally use three methods for damage assessment and imaging, such as the plane/focus swept method, full matrix capture (FMC), and total focusing method (TFM), a post-process technique [59,60]. The geometry of a general phased array is highlighted in Figure 1, while Figure 2 presents the scanning technique used. These B-scan techniques can then be performed along the surface of the structure (*y*-axis) in order to generate a C-scan image.

The TFM method uses a post-processing algorithm which discretises the thickness of a sample into a grid. The signals from all the elements in the array (referring to that grid point) are then summed to focus on every point in the grid. The intensity of the image, $I\left(x,z\right)$ at any point in the scan, is given by:

(1) $$I\left(x,z\right)=\left|\sum h_{tx,rx}\left(\frac{\sqrt{{\left(x_{tx}-x\right)}^{2}+z^{2}}+\sqrt{{\left(x_{rx}-x\right)}^{2}+z^{2}}}{c_{1}}\right)\right|\;,\;summed\;for\;\left|x_{tx,rx}-x\right|\leq \frac{D}{2}\;s$$

where: D is the width of the aperture; z and x are distances in the z-direction (normal to array, depth) and *x*-direction (position along array) for transmitting $\left(tx\right)$ and receiving elements $\left(rx\right)$ (see Figure 1); $h_{tx,rx}$ is the Hilbert transform of the time domain signal; $c_{1}$ is the speed of sound in the medium; s is the step along the array, which sets the resolution of the B-scan.

If an ultrasound array is considered and set up as in Figure 2a,b below, multiple k transmit and receive positions (pulse-echo testing) will result in multiple time-domain response signals for each point tested (A-scans). Multiple A-scans along a line (*x*-direction) can be used to generate a B-scan with all points (*x* and *y*-direction), generating a C-scan. The acquired data were post-processed by the authors using MATLAB software code.

Therefore, the described phased array system can acquire the back-scattered signals caused by several material variations, such as porosity, interfacial resin-rich areas, variations in fibre density, waviness in the plies, and others [61].

### 2.1. Subharmonic Method

The resonance of a bubble in a liquid is a well-studied phenomenon, with early works focusing on ultrasonic cavitation of bubbles within liquid mediums, where the amplitude and pressure of an ultrasonic pressure wave has been shown to either grow or collapse these bubbles under pressure. The oscillation of such bubbles in a liquid medium can be described using highly non-linear equations, such as the Rayleigh–Plesset equation [62,63]:

(2) $$R\ddot{R}+3\frac{{\dot{R}}^{2}}{2}=\left(\frac{1}{\rho }\right)\left\{\left(P_{0}+\frac{2\sigma }{R_{0}}-P_{v}\right){\left(\frac{R_{0}}{R}\right)}^{3\kappa }-\frac{2\sigma }{R}-\frac{4\mu \dot{R}}{R}-P_{0}-P\left(t\right)\right\},$$

where R is the bubble radius, $R_{0}$ its equilibrium value, σ surface tension, ρ density, μ viscosity of the liquid, $P_{0}$ hydrostatic pressure, $P\left(t\right)$ time-varying pressure component, $P_{v}$ vapour pressure within the bubble, and κ the polytropic index of the gas.

To approximate the resonant oscillations of an unforced bubble, viscosity and surface tension are assumed negligible, $P\left(t\right)$ is set to zero [29], and the frequency is deemed [64]:

(3) $$v_{r}=\left(\frac{1}{2\pi R_{0}}\right){\left(\frac{{3\kappa P}_{0}}{\rho }\right)}^{\frac{1}{2}}.$$

Although these formulations refer to bubbles in liquid mediums, they give insight into the resonant behaviour of entrapped gases in mediums, but also the porosity within the resin. Thus, it is possible to exploit the behaviour and ability of ultrasonic-excited porosity to generate subharmonic responses, as the principle behind the mechanism remains the same.

Furthermore, it is well-known that both micro-cracks and delamination, when excited by ultrasonic waves, can generate nonlinear material responses that can be analytically modelled using the classical nonlinear elasticity (CNE) theory [65]. Assuming a one-dimensional longitudinal wave propagation along the *x*-direction, the elastodynamic wave equation, as shown by Equation (1), can be expressed as the power series of the strain $\varepsilon_{x}=\frac{\partial u\left(x,t\right)}{\partial x}$ as follows:

(4) $$\rho \frac{\partial^{2}u\left(x,t\right)}{\partial x^{2}}=\frac{\partial \sigma }{\partial x}=\left(\lambda +2\mu \right)\left[\frac{\partial }{\partial x}\left(1+\beta \varepsilon_{x}+\gamma \varepsilon_{x}^{2}\right)\varepsilon_{x}\right]\;,$$

where σ is the stress, λ and μ are, respectively, Lamé’s first and second parameter, β and γ are the second and third order elastic coefficients, defined as the ratios between the second and third harmonic amplitudes and the square fundamental amplitude [39]. A normalised version of these parameters, which are only functions of the fundamental and high-order harmonic amplitudes, can be used as damage indicators because of their highest values at the damage location. The bispectral analysis is a valid alternative to the nonlinear coefficient’s methods, as it can be used to measure both the magnitude and phase of the higher-order harmonic frequency (second and third-order components) [42].

**Figure 3:**
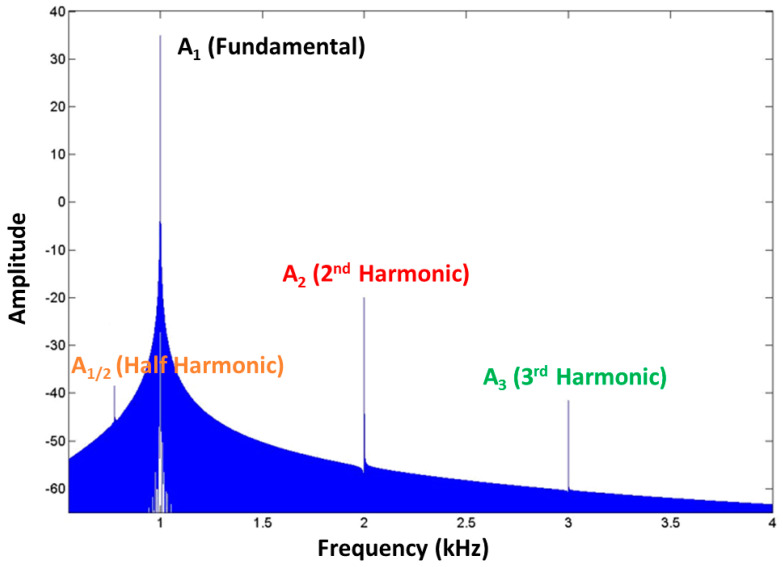
Generic amplitude spectrum (dB) of a backscattered signal with high-order and half-harmonic frequency components.

Nevertheless, out of the presented nonlinear methods, many research works demonstrated the benefit of subharmonic analysis when microbubbles, therefore porosity, are considered [53,65,66,67,68,69,70]. A generic amplitude spectrum of an acquired filtered backscattered signal can be presented as shown in Figure 3.

**Figure 4:**
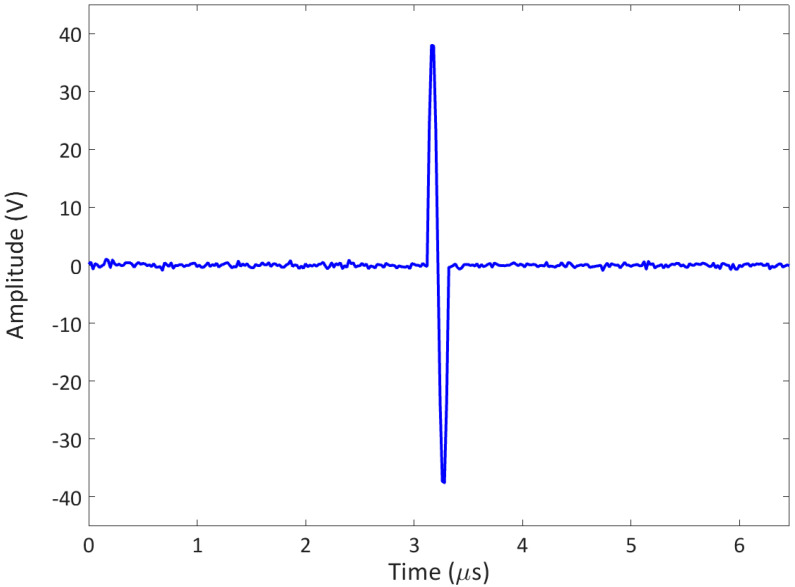
Input signal transmitted by the phased array system: 1-cycle tone burst.

Figure 3 shows the presence of both higher-order harmonics (at twice A2-2f1 and trice A3-3f1) and a half harmonic (f1/2), with the latter indicating the presence of microbubbles, such as porosity. The name is justified by the fact that this component is at half the fundamental frequency, which represents the frequency of the transmitted tone-burst signal (see Figure 4). Moreover, it is well-known that if a damaged sample is excited with a single frequency, the interaction between the generated waves and, for example, delamination produces contact acoustic nonlinearity in the form of higher harmonics, which provides a good measure for identifying the existence of delamination and determining their sizes in composites. Therefore, the spectrum presented in Figure 3 is due to the presence of both porosity and delamination.

**Figure 5:**
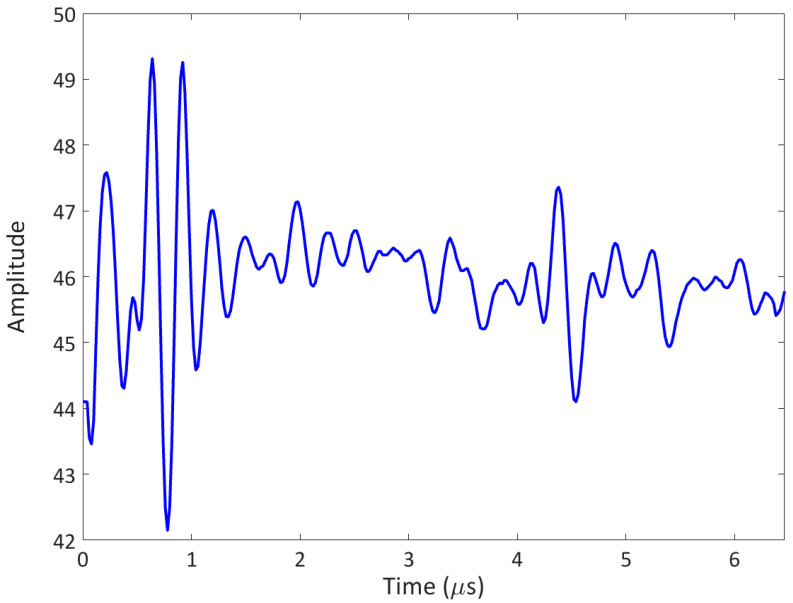
Output signal (arb. Amplitude) received by the phased array system: A-scan example.

In the experimental tests of this research work (see Section 4), only the contribution of the fundamental and the half-harmonic frequency components were considered since only microbubbles were present. The reliability of half-harmonic amplitudes of the received backscattered signals was noticeable (the A-scans, see an example in Figure 5) for the visualisation of the porosity field in the composite samples, identified by a 2D map, with each pixel representative of the extracted amplitude value of the half-harmonic component of a single backscattered signal.

### 2.2. Attenuation Coefficient Method

In Section 1, the relationship between the wave attenuation and the microscopic spheroidal dispersed voids within a composite sample was emphasised [15]. Therefore, a technique based on the attenuation coefficient was investigated and presented in this research work. This technique is based on a pulse-echo method achieved by a phased array system, as performed in the previous algorithm (see Section 2.1), with a 1-cycle tone burst as the transmitted signal (see Figure 4). If a single A-scan is considered and acquired, the amplitude spectrum $S\left(f\right)$ of the ultrasonic pulse is calculated. It should be noted that the spectrum can be written as:

(5) $$S\left(f\right)=S_{0}\left(f\right)\;exp\left[-\alpha \left(f\right)\;H\right]\;,$$

where $S_{0}\left(f\right)$ is the amplitude spectrum of the reference ultrasonic signal, H is the thickness of the specimen, and $\alpha \left(f\right)$ represents the attenuation coefficient function depending on the frequency that can be determined from Equation (5) as follows:

(6) αf=1H ln⁡S0fSf.

Once the attenuation coefficient function is obtained, it is possible to evaluate the porosity content in a composite sample since the proportionality between porosity and frequency are derivative of the attenuation coefficient function.

**Figure 6:**
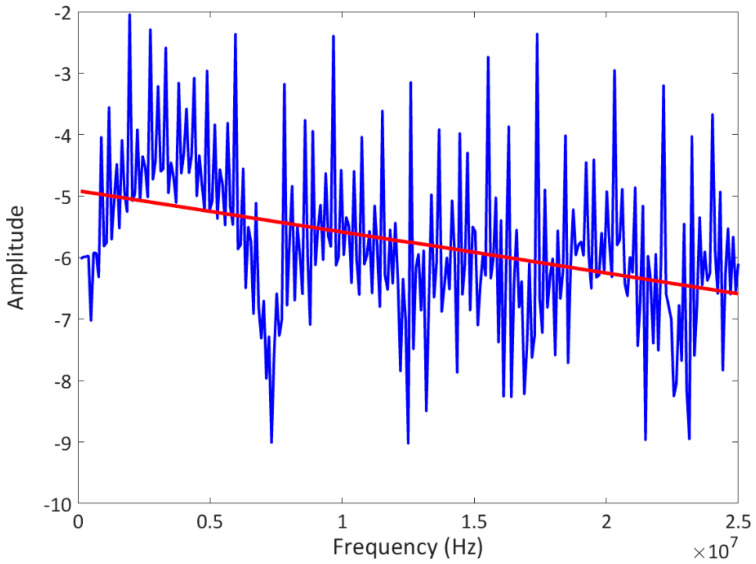
Attenuation coefficient in frequency domain and the linear trend, representative of the porosity amount.

Figure 6 shows an example of the attenuation coefficient calculated using Equation (6). The red line indicates the linear trend of the function, where the slope gradient is representative of the porosity details along a line from a single point of the specimen surface through the thickness. The application of the described algorithm to all the acquired A-scans leads to a 2D map showing the inner porosity field. Currently, the attenuation coefficient is generally used among other factors, such as frequency parameters, as a rapid tool to evaluate the structural quality of the composites [66].

## 3. Experimental Setup

**Figure 7:**
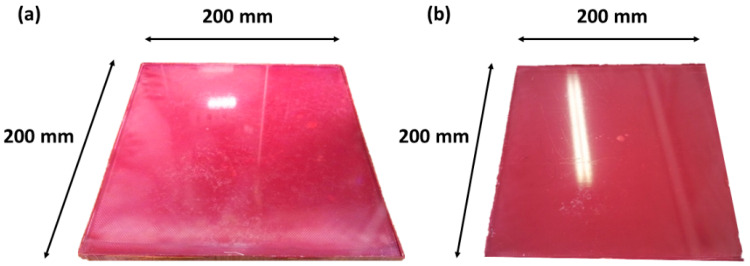
Porous composite sample 1 (**a**) and sample 2 (**b**).

In this study, two squares of composite samples, with dimensions of 200 mm × 200 mm × 9 mm and induced porosity due to manufacturing, were considered for the experimental tests (see Figure 7).

**Figure 8:**
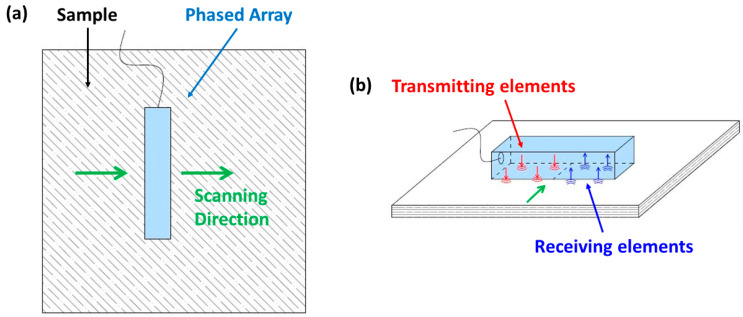
Phased array experimental system: top view (**a**) and ISO view (**b**).

The phased array system (Diagnostic Sonar—FI Toolbox) described in Section 2 (see Figure 8) utilised a 5 MHz 128-element probe (Diagnostic Sonar) with an element pitch of 0.5257 mm and width of 0.5 mm. A stepped linear (or sequential) C-scan routine was used to evaluate the porosity in the two samples. The step linear routine used beams of 32 elements, equating to a total scan width of ~58.8 mm. A half-cycle sine-wave tone burst with an amplitude of 80 V was used as the transmitting input signal. Due to the span of the array (~58.8 mm), four horizontal scanning stripes were necessary to cover the whole area of the two samples (see Figure 8). Raw data over the C-scan length were then post-processed according to the algorithms outlined in Section 2.1 and Section 2.2.

## 4. Results

This section illustrates the obtained results by using the algorithms described in Section 2.1 and Section 2.2. The results from the two developed methods are compared with classic C-scan results, which are regarded as the baseline and called “C-map”. The mentioned outputs consist of 2D maps of the three specimens, visualising the detected inner porosity field. The phased array probe was used to capture A-scans over the inspected area, and the subharmonic amplitude of each A-scan was used to build a 2D map, here named “S-map”. Likewise, another 2D map was created from the derivative, in the frequency domain, of the attenuation coefficient at each scan point. This map output is named “D-map”.

**Figure 9:**
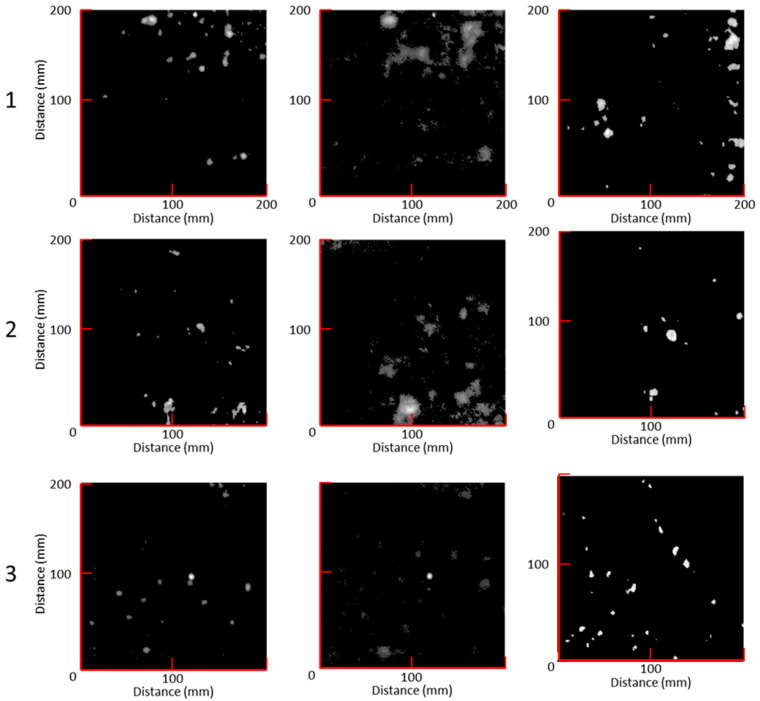
Porosity Imaging: experimental results.

In Figure 9, for each specimen, S-maps and D-maps are compared to the respective C-maps. The percentage *P* of the porous area with respect to the total area was calculated for each specimen as follows (see Table 1):

(7) $$P=\frac{{\sum A}_{bubble}}{A_{tot}}\times 100\%,$$

where *A_bubble_* is the detected 2D area of a single bubble, whilst *A_tot_* is the total area of the specimen.

According to Table 1, the S-maps showed more defects due to larger surface areas for identified porosity regions as well as the identification of smaller porosity, with an average of 405% higher detection than the C-Map. However, the D-maps provided a more uniform identification of each void, with an average of 194% higher detection than the C-Map. It should be noted that in each of the samples highlighted in Figure 9, there are areas of porosity that are clearly visible using one method but not using the other method. This reinforces the assumption that a combined approach, using multiple techniques with different advantages and disadvantages, increases the probability of detection.

## 5. Conclusions

This paper presented two nonlinear ultrasound techniques able to furnish a detailed 2D map of the porosity field within two undamaged samples. A pulse-echo method was performed by using a phased array system, able to transmit a tone-burst signal and receive the backscattered signal, representative of the sample content through the thickness. Data coming from all the sample points were post-processed, visualising the inner porosity by a reconstructed 2D map. A half-harmonic technique was implemented due to the microbubble peculiarity to emit a frequency equal to half the transmitted one [67,68,69,70]. A second method was based on the attenuation coefficient due to the relation between the wave attenuation and the presence of microscopic voids in a composite sample. Indeed, the frequency derivative of the attenuation coefficient is proportional to the porosity content in the specimen. The obtained results were compared, with a classic C-scan regarded as a baseline. All the obtained maps showed different levels and locations of inner porosity, with the S-Map and D-Map showing, on average, 405% and 194% more porosity in terms of area compared with the standard C-Map. While these results would suggest that the C-Map should be disregarded, this is not the case, as each of the methods provided useful and differing evaluations of the levels of porosity in the tested samples in terms of the location and size of the affected areas. Thus, it is suggested that the methods are used in tandem in order to maximise the probability of detection.

In order to maximise the probability of detection, further research is needed to combine the outputs from the various methods by developing algorithms to provide a single image of the levels of porosity.

## Figures and Tables

**Table 1:** Porosity area to total area ratios as a percentage, brackets relate to increase in relation to C-Maps.

Sample	C-Maps	S-Maps	D-Maps
1	2.26	9.17 (406%)	3.75 (166%)
2	1.4	7.99 (570%)	3.13 (224%)
3	1.13	2.7 (239%)	2.18 (193%)

## Data Availability

No data available.

## References

[1] Rubin A.M., Jerina K.L. (1994). Evaluation of porosity in composite aircraft structures. Mech. Compos. Mater..

[2] Stone D., Clarke B. (1975). Ultrasonic attenuation as a measure of void content in carbon-fibre reinforced plastics. Non Destr. Test..

[3] Bowles K.J., Frimpong S. (1992). Void Effects on the Interlaminar Shear Strength of Unidirectional Graphite-Fiber-Reinforced Composites. J. Compos. Mater..

[4] Müller de Almeida S.F., de Mas Santacreu A.C. (1995). Environmental effects in composite laminates with voids. Polym. Polym. Compos..

[5] Jeong H. (1997). Effects of Voids on the Mechanical Strength and Ultrasonic Attenuation of Laminated Composites. J. Compos. Mater..

[6] Suarez J.C., Molleda F., Guemes A. (1993). Void content in carbon fibre/epoxy resin composites and its effects on compressive properties. ICCM/9 Compos. Prop. Appl..

[7] de Almeida S.F.M., Neto Z.D.S.N. (1994). Effect of void content on the strength of composite laminates. Compos. Struct..

[8] Costa M.L., Almeida S.F.M., Rezende M.C. (2001). The influence of porosity on the interlaminar shear strength of carbon/epoxy and carbon/bismaleimide fabric laminates. Compos. Sci. Technol..

[9] Fernlund G., Wells J., Fahrang L., Kay J., Poursartip A. (2016). Causes and remedies for porosity in composite manufacturing. IOP Conference Series: Materials Science and Engineering.

[10] Martin B.G. (1977). Ultrasonic wave propagation in fiber-reinforced solids containing voids. J. Appl. Phys..

[11] Reynolds W.N., Wilkinson S.J. (1978). The analysis of fibre-reinforced porous composite materials by the measurement of ultrasonic wave velocities. Ultrasonics.

[12] Hsu D.K. (1988). Ultrasonic Measurements of Porosity in Woven Graphite Polyimide Composites. Review of Progress in Quantitative Nondestructive Evaluation.

[13] Nair S.M., Hsu D.K., Rose J.H. (1989). Porosity estimation using the frequency dependence of the ultrasonic attenuation. J. Nondestruct. Eval..

[14] Bhat M.R., Binoy M.P., Surya N.M., Murthy C.R.L., Engelbart R.W. (2012). Non-destructive evaluation of porosity and its effect on mechanical properties of carbon fiber reinforced polymer composite materials. AIP Conference Proceedings.

[15] Karabutov A.A., Podymova N.B. (2014). Quantitative analysis of the influence of voids and delaminations on acoustic attenuation in CFRP composites by the laser-ultrasonic spectroscopy method. Compos. Part B Eng..

[16] Pelivanov I., O’donnell M. (2015). Imaging of porosity in fiber-reinforced composites with a fiber-optic pump–probe laser-ultrasound system. Compos. Part A Appl. Sci. Manuf..

[17] Ishii Y., Biwa S., Kuraishi A. (2016). Influence of porosity on ultrasonic wave velocity, attenuation and interlaminar interface echoes in composite laminates: Finite element simulations and measurements. Compos. Struct..

[18] Stamopoulos A.G., Tserpes K.I., Prucha P., Vavrik D. (2016). Evaluation of porosity effects on the mechanical properties of carbon fiber-reinforced plastic unidirectional laminates by X-ray computed tomography and mechanical testing. J. Compos. Mater..

[19] Mayr G., Gresslehner K.H., Hendorfer G. (2017). Non-destructive testing procedure for porosity determination in carbon fibre reinforced plastics using pulsed thermography. Quant. Infrared Thermogr. J..

[20] (2006). Standard Test Method for Constituent Content of Composite Materials.

[21] Plessix B.D.P.D., Lefébure P., Boyard N., Le Corre S., Lefèvre N., Jacquemin F., Sobotka V., Du Roscoat S.R. (2019). In situ real-time 3D observation of porosity growth during composite part curing by ultra-fast synchrotron X-ray microtomography. J. Compos. Mater..

[22] Oromiehie E., Garbe U., Prusty B.G. (2020). Porosity analysis of carbon fibre-reinforced polymer laminates manufactured using automated fibre placement. J. Compos. Mater..

[23] Podymova N.B., Kalashnikov I.E., Bolotova L.K., Kobeleva L.I. (2019). Laser-ultrasonic nondestructive evaluation of porosity in particulate reinforced metal-matrix composites. Ultrasonics.

[24] Song P., Liu J., Liu L., Wang F., Sun X., Liu Z., Xu L. (2022). Contactless inspection of CFRP artificial disbonds using combined laser thermography and laser ultrasonics with optical microphone. Compos. Struct..

[25] Pelivanov I., Ambroziński Ł., Khomenko A., Koricho E.G., Cloud G.L., Haq M., O’Donnell M. (2016). High resolution imaging of impacted CFRP composites with a fiber-optic laser-ultrasound scanner. Photoacoustics.

[26] Ashwell D.G., Chauhan A.P. (1973). A study of ½-subharmonic oscillations by the method of harmonic balance. J. Sound Vib..

[27] Lauterborn W. (1976). Numerical investigation of nonlinear oscillations of gas bubbles in liquids. J. Acoust. Soc. Am..

[28] Leighton T.G., Lingard R.J., Walton A.J., Field J.E. (1991). Acoustic bubble sizing by combination of subharmonic emissions with imaging frequency. Ultrasonics.

[29] Sojahrood A.J., Earl R., Haghi H., Li Q., Porter T.M., Kolios M.C., Karshafian R. (2021). Nonlinear dynamics of acoustic bubbles excited by their pressure-dependent subharmonic resonance frequency: Influence of the pressure amplitude, frequency, encapsulation and multiple bubble interactions on oversaturation and enhancement of the subharmonic signal. Nonlinear Dyn..

[30] Dutta D., Sohn H., Harries K.A., Rizzo P. (2009). A nonlinear acoustic technique for crack detection in metallic structures. Struct. Health Monit..

[31] Fierro G.M., Ciampa F., Ginzburg D., Onder E., Meo M. (2015). Nonlinear ultrasound modelling and validation of fatigue damage. J. Sound Vib..

[32] Fierro G.P.M., Meo M. (2015). Residual fatigue life estimation using a nonlinear ultrasound modulation method. Smart Mater. Struct..

[33] Morris W.L., Buck O., Inman R.V. (1979). Acoustic harmonic generation due to fatigue damage in high-strength aluminum. J. Appl. Phys..

[34] Donskoy D.M., Sutin A.M. (1998). Vibro-Acoustic Modulation Nondestructive Evaluation Technique. J. Intell. Mater. Syst. Struct..

[35] Cantrell J.H., Yost W.T. (2001). Nonlinear ultrasonic characterization of fatigue microstructures. Int. J. Fatigue.

[36] Hillis A.J., Neild S.A., Drinkwater B.W., Wilcox P.D. (2006). Global crack detection using bispectral analysis. Proceedings of the Royal Society of London A: Mathematical, Physical and Engineering Sciences.

[37] Boccardi S., Calla D., Fierro G.-P., Ciampa F., Meo M. (2015). Nonlinear Damage Detection and Localisation Using a Time Domain Approach. Struct. Health Monit..

[38] Fierro G.P.M., Calla’ D., Ginzburg D., Ciampa F., Meo M. (2017). Nonlinear ultrasonic stimulated thermography for damage assessment in isotropic fatigued structures. J. Sound Vib..

[39] Boccardi S., Callá D., Ciampa F., Meo M. (2018). Nonlinear elastic multi-path reciprocal method for damage localisation in composite materials. Ultrasonics.

[40] Meo M., Zumpano G. (2005). Nonlinear elastic wave spectroscopy identification of impact damage on a sandwich plate. Compos. Struct..

[41] Scalerandi M., Gliozzi A.S., Bruno C.L.E., Masera D., Bocca P. (2008). A scaling method to enhance detection of a nonlinear elastic response. Appl. Phys. Lett..

[42] Ciampa F., Pickering S., Scarselli G., Meo M. (2014). Nonlinear damage detection in composite structures using bispectral analysis. Health Monitoring of Structural and Biological Systems.

[43] Van Den Abeele K.A., Johnson P.A., Sutin A. (2000). Nonlinear elastic wave spectroscopy (NEWS) techniques to discern material damage, part I: Nonlinear wave modulation spectroscopy (NWMS). J. Res. Nondestruct. Eval..

[44] Straka L., Yagodzinskyy Y., Landa M., Hänninen H. (2008). Detection of structural damage of aluminum alloy 6082 using elastic wave modulation spectroscopy. NDT E Int..

[45] Fierro G.P.M., Meo M. Identification of the Location and Level of Loosening in a Multi-bolt Structure using Nonlinear Ultrasound. Proceedings of the 11th International Workshop on Structural Health Monitoring 2017: Real-Time Material State Awareness and Data-Driven Safety Assurance, IWSHM 2017.

[46] Fierro G.P.M., Meo M. (2016). Nonlinear imaging (NIM) of flaws in a complex composite stiffened panel using a constructive nonlinear array (CNA) technique. Ultrasonics.

[47] Fierro G.-P.M., Pinto F., Iacono S.D., Martone A., Amendola E., Meo M. (2017). Monitoring of self-healing composites: A nonlinear ultrasound approach. Smart Mater. Struct..

[48] Dionysopoulos D., Fierro G.-P.M., Meo M., Ciampa F. (2018). Imaging of barely visible impact damage on a composite panel using nonlinear wave modulation thermography. NDT E Int..

[49] Dos Santos S., Prevorovsky Z. (2011). Imaging of human tooth using ultrasound based chirp-coded nonlinear time reversal acoustics. Ultrasonics.

[50] Ciampa F., Meo M. (2012). Nonlinear elastic imaging using reciprocal time reversal and third order symmetry analysis. J. Acoust. Soc. Am..

[51] Zumpano G., Meo M. (2006). A new damage detection technique based on wave propagation for rails. Int. J. Solids Struct..

[52] Amerini F., Barbieri E., Meo M., Polimeno U. (2010). Detecting loosening/tightening of clamped structures using nonlinear vibration techniques. Smart Mater. Struct..

[53] Harput S., Arif M., Freear S. Experimental investigation of the subharmonic emission from microbubbles using linear and nonlinear frequency modulated signals. Proceedings of the 2010 IEEE International Ultrasonics Symposium.

[54] Cantrell J.H. (2003). Fundamentals and Applications of Nonlinear Ultrasonic Nondestructive Evaluation. Ultrasonic Nondestructive Evaluation.

[55] Ciampa F., Onder E., Barbieri E., Meo M. (2014). Detection and Modelling of Nonlinear Elastic Response in Damaged Composite Structures. J. Nondestruct. Eval..

[56] Ohara Y., Mihara T., Sasaki R., Ogata T., Yamamoto S., Kishimoto Y., Yamanaka K. (2007). Imaging of closed cracks using nonlinear response of elastic waves at subharmonic frequency. Appl. Phys. Lett..

[57] Park C.-S., Kim J.-W., Cho S., Seo D.-C. (2016). A high resolution approach for nonlinear sub-harmonic imaging. NDT E Int..

[58] Potter J.N., Croxford A.J., Wilcox P.D. (2014). Nonlinear Ultrasonic Phased Array Imaging. Phys. Rev. Lett..

[59] Oralkan O., Ergun A.S., Johnson J.A., Karaman M., Demirci U., Kaviani K., Lee T., Khuri-Yakub B.T. (2002). Capacitive micromachined ultrasonic transducers: Next-generation arrays for acoustic imaging?. IEEE Trans. Ultrason. Ferroelectr. Freq. Control.

[60] Chiao R.Y., Thomas L.J. (1994). Analytic evaluation of sampled aperture ultrasonic imaging techniques for NDE. IEEE Trans. Ultrason. Ferroelectr. Freq. Control.

[61] Kim K.-B., Hsu D.K., Barnard D.J. (2013). Estimation of porosity content of composite materials by applying discrete wavelet transform to ultrasonic backscattered signal. NDT E Int..

[62] Neppiras E.A., Noltingk B.E. (1951). Cavitation Produced by Ultrasonics: Theoretical Conditions for the Onset of Cavitation. Proc. Phys. Soc. Sect. B.

[63] Neppiras E.A. (1980). Acoustic cavitation. Phys. Rep..

[64] Minnaert M. (1933). On musical air-bubbles and the sounds of running water. Lond. Edinb. Dublin Philos. Mag. J. Sci..

[65] Ostrovsky L.A., Johnson P.A. (2001). Dynamic nonlinear elasticity in geomaterials. Riv. Nuovo Cimento.

[66] Karabutov A.A., Podymova N.B., Belyaev I.O. (2013). The influence of porosity on ultrasound attenuation in carbon fiber reinforced plastic composites using the laser-ultrasound spectroscopy. Acoust. Phys..

[67] Eller A., Flynn H.G. (1969). Generation of Subharmonics of Order One-Half by Bubbles in a Sound Field. J. Acoust. Soc. Am..

[68] Krishna P.D., Shankar P.M., Newhouse V.L. (1999). Subharmonic generation from ultrasonic contrast agents. Phys. Med. Biol..

[69] Forsberg F., Shi W.T., Goldberg B.B. (2000). Subharmonic imaging of contrast agents. Ultrasonics.

[70] Biagi E., Breschi L., Vannacci E., Masotti L. (2006). Subharmonic emissions from microbubbles: Effect of the driving pulse shape. IEEE Trans. Ultrason. Ferroelectr. Freq. Control.

